# Supplementary material for: A Fructan Exohydrolase from Maize Degrades Both Inulin and Levan and Co-Exists with 1-Kestotriose in Maize
Source: Int J Mol Sci. 2021 May 13;22(10):5149. doi: 10.3390/ijms22105149 (PMC8152283; doi:10.3390/ijms22105149)
Supplement: Supplementary file 1 [file ijms-22-05149-s001.zip › ijms-1199746-supplementary.pdf]

International Journal of
Molecular Sciences

MDPI

Supplementary Materials:

Table S1. The Predicted Subcellular Localization of Zm-6&1-FEH2.

Program	Apoplast	ER	Golgi	CHL	MT	Other
PSORT	0.814[a]	0.2	NA	NA	NA	0.178
TargetP	0.274	NA	NA	0.141	0.377	0.018
SignalP	0.923[a]	NA	NA	NA	NA	NA

The three intracellular targeting prediction programs used were PSORT (http://psort.hgc.jp/), Target P (http://www.cbs.dtu.dk/services/TargetP/), and SIGNAL P (http://www.cbs.dtu.dk/ Services/SignalP/). CHL, chloroplast; ER, endoplasmic reticulum; Golgi, Golgi body; MT, mitochondria; NA, not applicable. a *The higher the value, the higher the probability of localization in the indicated subcellular compartment.*

Table S2. Oligonucleotides used for PCR amplification and cloning. Included are primers for full length constructs (full); partial cDNAs for qPCR (qPCR); for heterologous expression of mature proteins for *P. pastoris* overexpression (pPICZ).

cDNA	Comment	Primer name	Primer sequence (5'- 3')
Zm-6&1-FEH2	Full gateway +/-Stop codon	FEHGw-F	GGGGACAAGTTTGTACAAAAAAGCAGGCTCCATGGCCATGGC CATGGCCCGA
		FEHGw-R+	GGGGACCACTTTGTACAAGAAAGCTGGGTCTCATCCCTTGTTA ACCTGTGC
		FEHGw-R-	GGGGACCACTTTGTACAAGAAAGCTGGGTCTCCCTTGTTAACC TGTGC
	qPCR	FEHq-F	GCATGTACGCGTTCAACAAT
		FEHq-R	TAATTCATGTGCCCATGCTC
	pPICZαA	FEHpP-F	GACTAGTCTAGACCTCCCTTGTTAACCTGTGC
		FEHpP-R	GACTAGTCTAGACCCAGTGGATCCGCACGGTCGTC
Zm-INVINH1	Full gateway -Stop codon	Inh1Gw-F	GGGGACAAGTTTGTACAAAAAAGCAGGCTCCATGAAGCTTCT GCAAGCTCTGT
		Inh1Gw-R-	GGGGACCACTTTGTACAAGAAAGCTGGGTCCAACGCGGCCGT TACAGACAGC
Zm-ubiquitin	qPCR	UbiqPCR-F	GCTCTGACACCATCGACAACGTGAA
		UbiqPCR-R	GCTGCTTGCCGGCGAAGATC
Zm-β-tubulin	qPCR	TubuqPCR-F	CCTATAACGCCACGCTCTCTGT
		TubuqPCR-R	CATTGTCCAGCACCATGCA
